# Bilateral Discoid Medial Menisci With a Parameniscal Cyst: A Case Report

**DOI:** 10.1002/ccr3.71918

**Published:** 2026-02-03

**Authors:** Jean Michel Hovsepian, Johannes Glaser, André Lança Alves, Rafael José Melo Cué, Thilo Schmitt, Stephan Vogt

**Affiliations:** ^1^ Department of Orthopedic Sports Medicine and Arthroscopic Surgery Hessing Stiftung Augsburg Germany

**Keywords:** adolescent, arthroscopy, discoid medial meniscus, parameniscal ganglion, saucerization

## Abstract

Bilateral discoid medial menisci are an exceptionally rare anatomical variant, and their coexistence with a parameniscal cyst has only been described in isolated cases. We present the case of a 13‐year‐old male with activity‐related medial knee pain initially affecting the right knee. Imaging confirmed an incomplete discoid medial meniscus associated with a parameniscal cyst, which was treated with arthroscopy, open cyst excision, and meniscocapsular repair. A subsequent symptomatic retear required revision saucerization and combined meniscal fixation techniques. Months later, the patient developed similar symptoms in the contralateral knee, where MRI revealed a discoid medial meniscus with a horizontal tear, successfully treated with saucerization and partial meniscectomy. At final follow‐up, the patient remained pain‐free and fully active. This case highlights the importance of early recognition and preservation‐focused surgical treatment for this rare bilateral presentation.

## Key Clinical Message

Bilateral discoid medial menisci are extremely rare and may present with nonspecific symptoms in young athletes. Early recognition and meniscal preservation with saucerization and repair can successfully restore knee function and prevent long‐term complications.

## Graphical abstract

Rare bilateral discoid medial menisci with parameniscal cyst: clinical presentation, imaging, and arthroscopic management

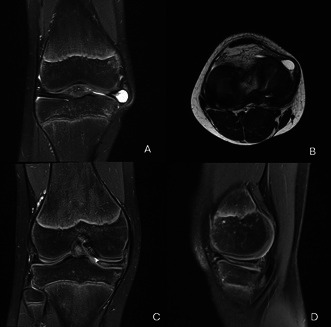

## 1 Introduction

A discoid meniscus represents an anatomical variation where the meniscus exhibits an atypical morphology, appearing as a complete circle or a semi‐circle, in contrast to the typical crescent moon shape. Given its abnormal ultrastructural and morphological characteristics, which include altered vascularization, atypical cellular distribution and shape, and disorganization of collagen fibers, the discoid meniscus possesses an inherent predisposition toward degeneration, injury, and meniscal tearing [1]. The incidence of discoid lateral meniscus ranges from 0.4% to 17%; in contrast, the discoid medial meniscus is considerably rare, with an incidence between 0.1% and 0.3% [2].

The first two documented true discoid medial meniscus (DMM) were reported in a six case‐series by Cave and Staples in 1941 [3] and it was not until 1956 when the first bilateral discoid medial meniscus (BDMM) occurrence documented by Murdoch et al. occurred [4]. The exact incidence of BDMM is extremely hard to determine due to its rarity, but it is estimated to range between 0.0056% and 0.012% [5]. Other pathologies, including the formation of cyst abnormalities, are associated with a discoid medial meniscus [6, 7]. To our knowledge the incidence of combined BDMM and cyst formation is unknown.

Early treatment for BDMM is essential for proper long‐term recovery and for preventing subsequent complications. Unfortunately, this is frequently delayed, especially in pediatric patients, because the condition's low incidence makes it hard to identify. Furthermore, the presence of ambiguous symptoms and the absence of trauma history in young patients further hinders prompt diagnosis [8].

We report a case of a young male patient with BDMM who was successfully treated at our institution.

## 2 Case History

In October 2023, a 13‐year‐old male patient presented to our sport orthopedics outpatient clinic in the company of his parents, referring recurring pain in his right knee, which worsened with exercise and interfered with his physical activity. The patient played handball frequently and reported that 2 years ago he suffered a blunt trauma to the knee, and a year ago he experienced pain and swelling in the knee following physical exertion.

On physical examination, mild genu valgum was documented bilaterally; swelling was visible on the medial aspect of the right knee when lying down. Range of motion 140°‐0°‐0° flexion/extension, equal on both sides with stable knee ligaments. Clinically mild medial meniscus signs were observed when the knee was flexed until 90°.

**FIGURE 1:**
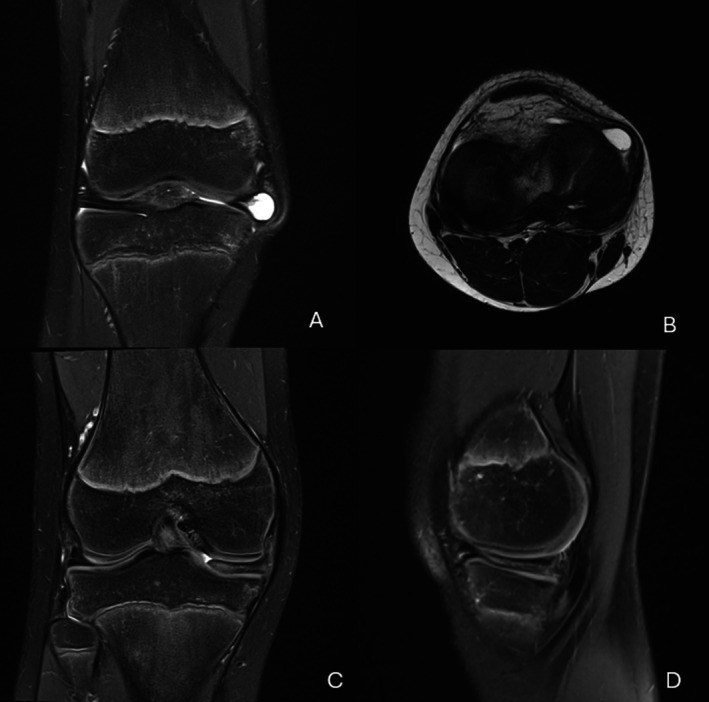
MRI of the right knee. The coronal T2‐weighted scan shows a parameniscal cystic mass from the medial meniscus (A) The axial T2 shows the parameniscal cystic lesion protruding from the junction of the pars intermedia and the anterior horn, extending through the medial retinaculum at the junction of the medial collateral ligament (B) Both the coronal and sagittal scans show the medial discoid meniscus (C, D).

Radiographs of the knee showed no remarkable findings. Magnetic resonance imaging (MRI) of the right knee demonstrated the presence of a medial discoid meniscus and a parameniscal cyst with high signal intensity at the junction of the pars intermedia and the anterior horn, indicative of potential intrameniscal degeneration (Figure 1).

**FIGURE 2:**
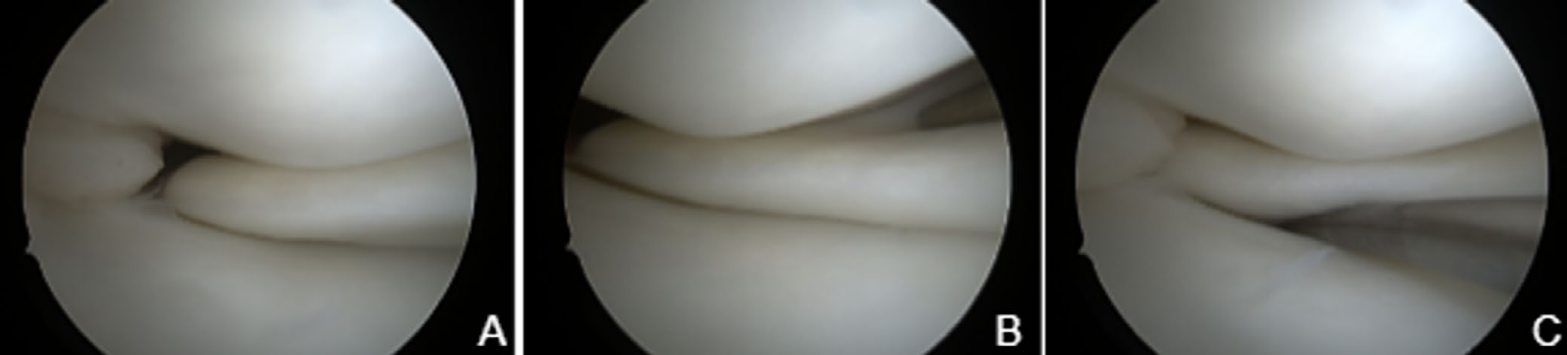
Right knee arthroscopic view of an incomplete medial discoid meniscus (A), stability test with the probe (B, C).

The patient underwent arthroscopy of the right knee, visualizing an incomplete medial discoid meniscus without signs of instability (Figure 2). The parameniscal ganglion was completely removed with an open approach and sent for histologic evaluation. The meniscocapsular layer was repaired with multiple vertical PDS sutures.

**FIGURE 3:**
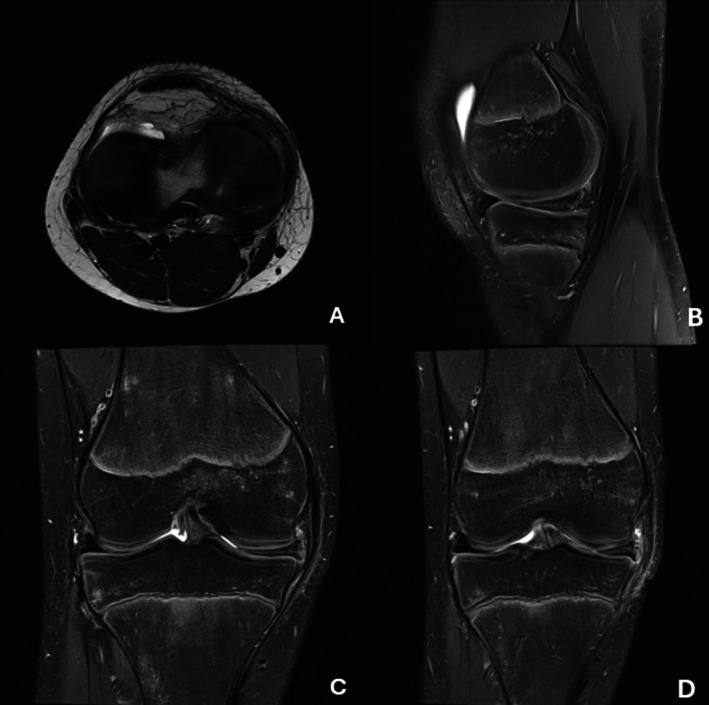
MRI of the right knee. The axial T2‐weighted scan shows a vertical tear at the base of the posterior horn (A). The sagittal T2 scan shows a high signal intensity in the medial meniscus, indicative of a vertical tear (B). Coronal view shows a horizontal cleavage tear in the medial meniscus (C, D).

The patient remained asymptomatic at 4‐ and 6‐week follow‐ups. At the 12‐week postoperative visit, a locking and clicking sensation was reported during full knee extension. Physical examination revealed a palpable clicking sensation along the medial joint line during terminal knee extension (0°–10°), indicative of medial meniscal instability. Postoperative MRI demonstrated a new vertical tear at the base of the posterior horn, potentially accounting for the observed meniscal instability (Figure 3).

**FIGURE 4:**
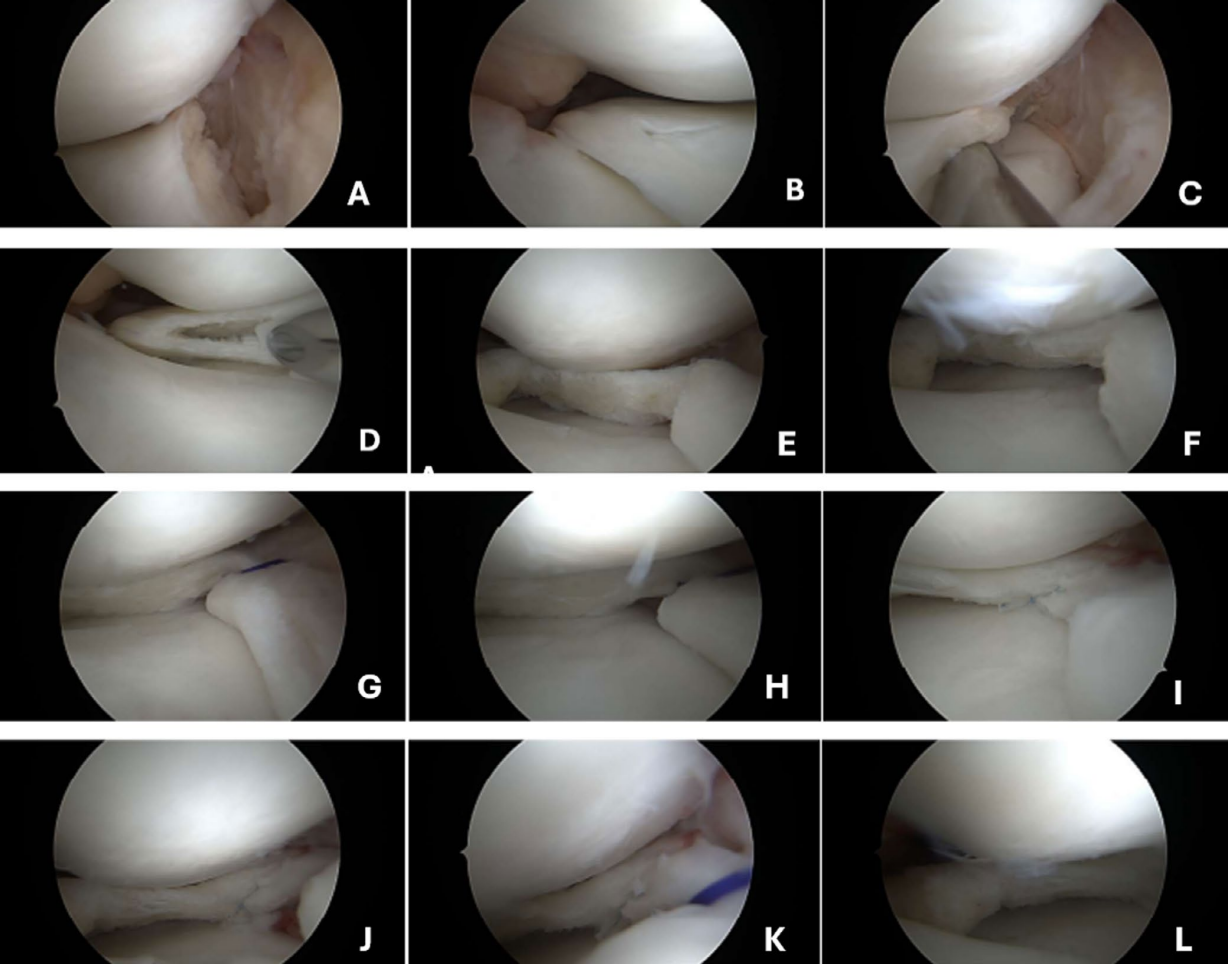
Right knee arthroscopic images showing a tear and dehiscence in the capsulomeniscal junction of the medial meniscus (A–C), a horizontal cleavage tear in the posterior horn of the medial meniscus (D), images after debridement of the medial meniscus (E, F), repair of the medial meniscus with an all‐inside technique and outside‐in suture (G–L).

The patient underwent arthroscopic surgery a second time. In the medial compartment, a vertical tear was identified at the meniscocapsular junction, demonstrating instability extending from the midbody toward the anterior horn of the medial meniscus. After saucerization of the discoid meniscus, a horizontal cleavage tear was identified in the posterior horn. Following debridement of the meniscus and the meniscocapsular region, meniscal repair was performed. The first suture was placed using an all‐inside repair technique (Fast‐Fix 360°, Smith & Nephew) at the junction between the posterior horn and the midbody. Subsequently, an additional suture was placed using an outside‐in technique with a PDS suture to stabilize the midbody (Figure 4). At the latest follow‐up (20 months postoperatively), the patient remained asymptomatic, with a satisfactory clinical outcome. Follow‐up MRI demonstrated no evidence of meniscal instability or other abnormal findings. Postoperative functional assessment showed improvement, with Lysholm and IKDC scores of 91 points and 76%, respectively.

**FIGURE 5:**
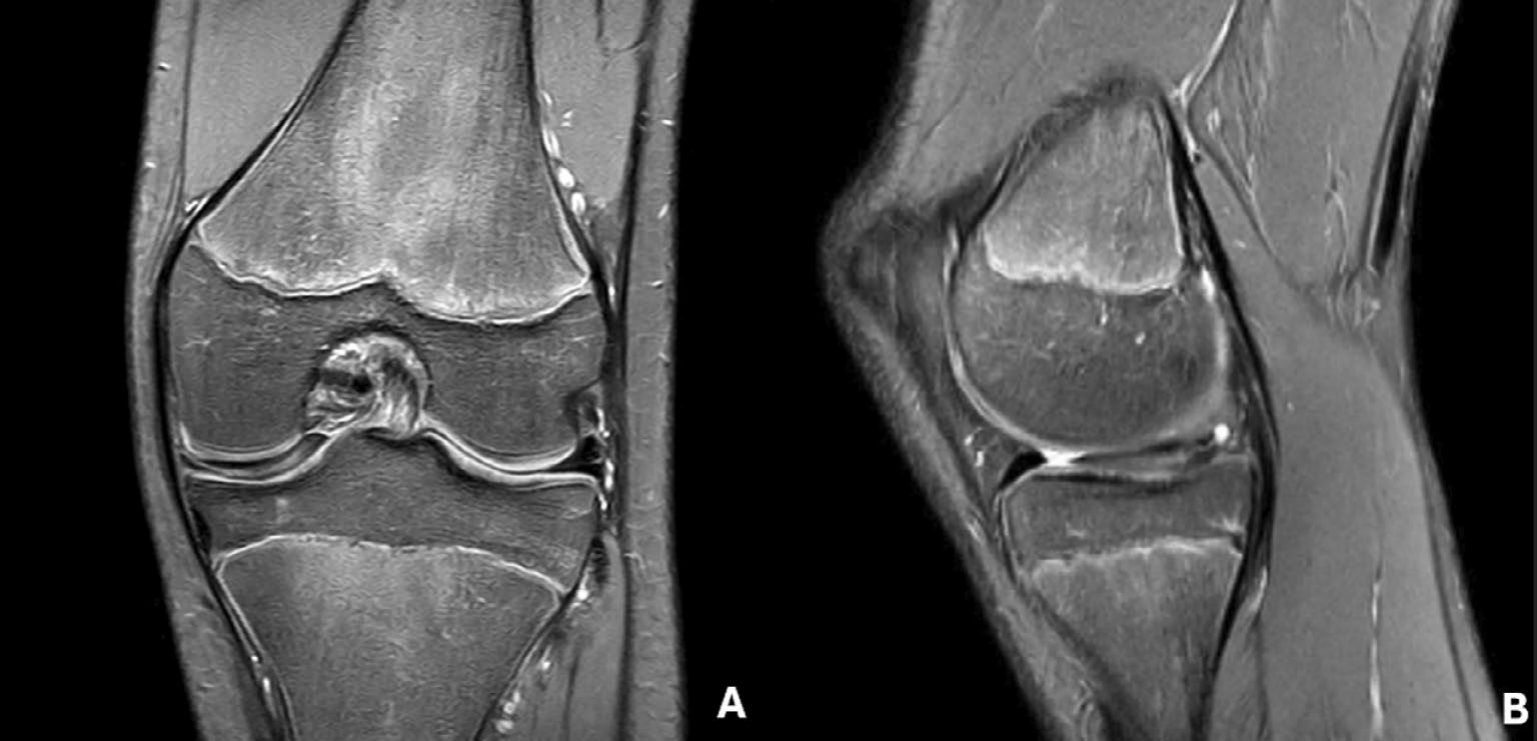
MRI of the left Knee. The coronal T2‐weighted scan shows a discoid medial meniscus, along with a degenerative horizontal cleavage tear (A) The tear extends peripherally dorsomedial in the area of the junction of the posterior horn and the corpus fossa, contacting the base with a parameniscal cyst of 3 mm as well as the surface with incipient displacement of both meniscal layers (B).

Nine months after the revision surgery, the patient presented for follow‐up, reporting no symptoms in the right knee. Moreover, he described symptoms similar to the initial presentation, now affecting the contralateral knee, characterized by persistent, rotation‐dependent pain, particularly during deep flexion and while sitting cross‐legged. He stated that pain‐free physical activity was no longer possible. MRI of the left knee also revealed a discoid configuration of the medial meniscus with mucoid degeneration and a long horizontal tear (Figure 5).

**FIGURE 6:**
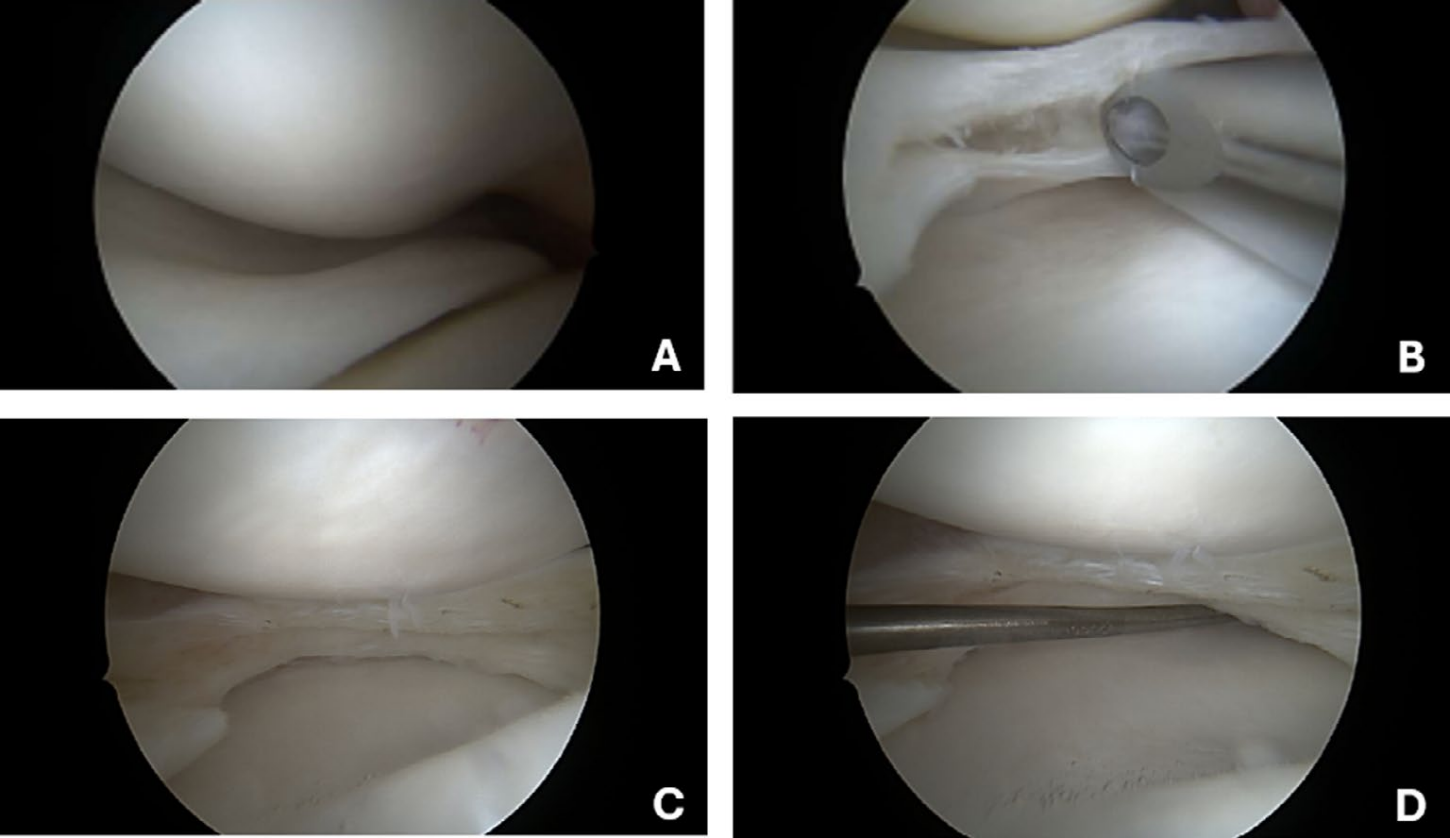
Left Knee arthroscopic view of medial discoid meniscus (A) degenerative horizontal cleavage tears on the medial meniscus (B) stability test with the probes (C, D).

Arthroscopic surgery was recommended for the left knee, consisting of saucerization and debridement of the discoid meniscus (Figure 6). The procedure was performed with satisfactory intraoperative and postoperative results. The patient remained asymptomatic during the 8‐month follow‐up period and returned to his daily activities without any limitations after 12 months. No recurrence of the parameniscal ganglion was observed on the right side.

## 3 Discussion

We documented the case of a young man treated for BDMM, which treatment involved open cystectomy and suturing of the discoid meniscus. A subsequent revision procedure included saucerization and fixation on the right side, and partial meniscectomy with saucerization on the left.

Treatment for discoid meniscus has been described in several studies for the lateral side [2, 9, 10, 11]. Patients without symptoms can be treated conservatively [2]. For symptomatic patients, treatment options consist of partial meniscectomy with saucerization of the discoid meniscus with or without meniscal repair [2, 9, 11, 12]. Ahn et al. reported favorable functional results and improved Lysholm Scores for combined partial meniscectomy and meniscal repair for peripheral tears in discoid lateral meniscus after an average follow‐up of 51 months [9]. These treatment options are commonly used for the medial discoid meniscus. For symptomatic discoid medial meniscus treatment was reported in several studies with saucerization with or without meniscal repair [5, 13, 14, 15, 16, 17], mostly case reports. Feroe et al. described a collective of eleven knees and performed eleven saucerization's with medial meniscus repair in seven. They found retears in 36% at a mean of 25, 8 months postoperatively [14].

Due to the low incidence of DMM, there are no studies available for examining treatment options for DMM accompanying meniscal cysts. For normal meniscus, open or arthroscopic cyst resection together with meniscus repair or partial resection are current treatment options [18]. As arthroscopic treatment of the cyst is associated with higher recurrence rates, open cyst resection should be considered, especially for bigger cysts [18].

It was reported in two cases that BDMM was associated with cyst formation [6, 7]. Histological examination has shown reduced quality of collagen fibers [2]. This might be a reason for the appearance of ganglion cysts. Franceschi et al. reported a case with BDMM with a posterior horn cyst of the symptomatic DMM. Three years after arthroscopic meniscectomy and cyst resection the patient had no symptoms and no pain [6]. Kim et al. reported a case with BDMM with a cyst formation on the anterior portion of a symptomatic DMM with an anomalous insertion to the anterior cruciate ligament. Six months after partial meniscectomy and arthroscopic cyst resection the patient was pain free with good functional outcome. On the asymptomatic contralateral side a MRI scan showed a DMM with anomalous insertion to the ACL, but no cyst formation [7].

## 4 Conclusion

Arthroscopic partial meniscectomy, saucerization, and fixation is a current treatment strategy for DMM. Concomitant cysts should be treated either by open or arthroscopic cystectomy. More studies are needed to compare different treatments and examine long‐term outcomes.

## Author Contributions

**Jean Michel Hovsepian:** conceptualization, data curation, formal analysis, investigation, methodology, supervision, writing – review and editing. **Johannes Glaser:** formal analysis, writing – original draft. **André Lança Alves:** data curation, methodology, writing – original draft. **Rafael José Melo Cué:** data curation, formal analysis, methodology, writing – original draft. **Thilo Schmitt:** validation, writing – review and editing. **Stephan Vogt:** conceptualization, supervision, validation, visualization, writing – review and editing.

## Funding

The authors have nothing to report.

## Consent

Written informed consent was obtained from the patient and the patient's legal guardian for publication of this case report.

## Conflicts of Interest

The authors declare no conflicts of interest.

## Data Availability

The data supporting the findings of this study are available from the corresponding author upon reasonable request.
